# Molecular cloning of *SLC35D3* and analysis of its role during porcine intramuscular preadipocyte differentiation

**DOI:** 10.1186/s12863-020-0822-0

**Published:** 2020-02-22

**Authors:** Wentong Li, Keliang Wu, Ying Liu, Yalan Yang, Wenwen Wang, Xiuxiu Li, Yanmin Zhang, Qin Zhang, Rong Zhou, Hui Tang

**Affiliations:** 1 Shandong Provincial Key Laboratory of Animal Biotechnology and Disease Control and Prevention, College of Animal Science and Technology, Shandong Agricultural University, 61 Daizong Street, Tai’an, 271018 People’s Republic of China; 2 The State Key Laboratory of Animal Nutrition, Institute of Animal Science, Chinese Academy of Agricultural Sciences, Beijing, 100193 People’s Republic of China; 3 College of Animal Science and Technology, China Agricultural University, Beijing, 100193 People’s Republic of China; 4 School of Life Science and Engineering, Foshan University, Foshan, 528231 Guangdong China

**Keywords:** *SLC35D3*, cDNA clone, Sequence characteristics, Tissue expression, Preadipocytes

## Abstract

**Background:**

Solute carrier family 35 (SLC35) is one of a large number of membrane transporter protein families. Member D3 of this family is thought to be involved in adipose deposition and metabolic control.

**Results:**

We obtained 2238 bp cDNA of porcine *SLC35D3*, it contains a 1272 bp ORF, encoding a 423 amino acid polypeptide, and a 966 bp 3′ UTR. BLAST results revealed that the amino acid sequence of porcine SLC35D3 had the closest phylogenetic relationship with members of the genus *Ovis aries*. Further bioinformatics analysis showed that the SLC35D3 protein contains 8 transmembrane domains, and that there is no signal peptide structure. The secondary structure of the protein mainly contains 37.12% α-helixes, 7.8% in β-folds, and 33.57% random coils. mRNA expression analysis showed that *SLC35D3* is expressed in lung, liver, heart, spleen, kidney, longissimus dorsi muscle (LDM), leaf fat (LF), and subcutaneous adipose tissue (SAT). To examine the effects of *SLC35D3* expression on fat synthesis and catabolism, *SLC35D3*-siRNA was transfected into cultured intramuscular adipocytes. *SLC35D3* silenced cells showed increased expression of genes related to fat synthesis, and increased deposition of intramuscular fat (IMF), abundance of lipid droplets, and the level of free fatty acid (FFA) in the culture medium. In contrast, the siRNA decreased the expression genes involved in fat catabolism.

**Conclusions:**

Our results demonstrate that silenced *SLC35D3* results in increased adipogenic processes in pig intramuscular adipocytes. These data represent the first exploration of *SLC35D3* expression in swine, and provide valuable insights into the functions of *SLC35D3* in adipocyte differentiation.

## Background

Pigs have long served as models in biomedical research because of their similarity to humans with regard to body size, physiological conditions, eating patterns, and fat deposition [1–4]. Pig breeds do vary in fat deposition and are characterized by differences in intramuscular fat content and backfat thickness. Some indigenous Chinese breeds, such as the Yimeng Black pig, exhibit particularly high body fat mass [5, 6]. Excessive fat deposition, which can result in obesity and disorders of energy metabolism in humans, is an important predictor of metabolic abnormalities [7]. Thus, the pig is an ideal model for the study of obesity and metabolic syndrome (MetS) [8, 9].

Obesity is a complex disease, it is influenced by genetic, environmental, and phenotypic factors [10–12], but the underlying mechanisms are not well understood [13]. Obesity is mainly determined by genetic differences [14], thus identification of the genes involved in fat deposition is of great interest. The solute carrier (SLC) group is the second largest group of membrane transport proteins, with more than 400 members in more than 60 families [15]. These proteins participate in numerous physiological processes including the transporting of inorganic ions, amino acids, sugars, lipids, neurotransmitters, and drugs [15]. Human *SLC35D3* is associated with fat deposition and is a candidate gene for MetS [16]. *SLC35D3* is also involved in the biogenesis of platelet dense granules, and its expression in the brain is limited to the expression of dopamine receptor D1, though not receptor D2 [16–18]. Other research indicates that *SLC35D3* is an important regulator of tissue-specific autophagy [19]. *SLC35D3* therefore offers an opportunity to understand the mechanisms of fat deposition, and may lead to therapeutic interventions for obesity.

In this study, we cloned the *SLC35D3* CDS from the Yimeng Black pig, analyzed its amino acid sequence, and studied its expression in selected tissues and organs. We then transfected cultured intramuscular adipocytes with an *SLC35D3*-siRNA and examined the cells for changes in fatty acid metabolism. The results establish a foundation for understanding the function of the *SLC35D3* gene in pigs.

## Results

### Cloning and sequence analysis of the *SLC35D3* gene

**Fig. 1:**
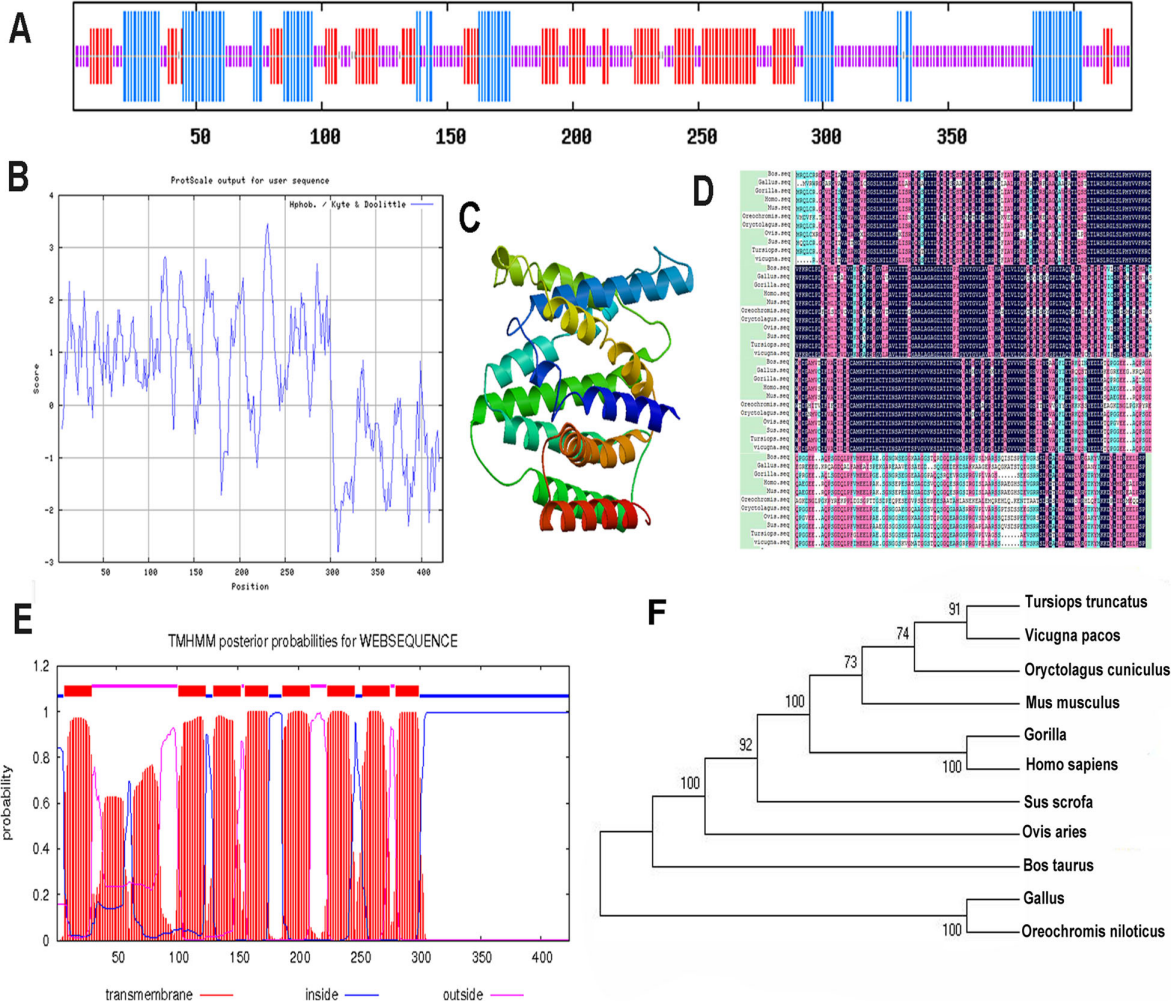
Sequence analysis of *Sus scrofa* SLC35D3. **a** Predicted secondary structure of the porcine SLC35D3 amino acid sequence. The blue lines represent α-helixes, red lines represent extended strands, and purple lines represent random coils. **b** Hydrophobicity profile of *Sus scrofa* SLC35D3 protein. The y-axis displays hydrophilic index; the x-axis displays the amino acid position. **c** Predicted tertiary structure of SLC35D3. **d** Multiple sequence alignment of the deduced amino acid of SLC35D3. **e** Predicted transmembrane domains of SLC35D3. **f** Phylogenetic tree of SLC35D3 amino acid sequences from 11 organisms, constructed using the neighbor-joining method

Although the sequence of the porcine *SLC35D3* was predicted and annotated within the *Sus scrofa* genome (NC_010443.4), a cDNA version has not yet been cloned. To obtain a full-length cDNA, we designed PCR primers using the annotated sequence, and used total RNA extracted from the liver tissue of a Yimeng Black pig as a PCR template. The PCR product was cloned and sequenced, the fragment (2238 bp) (KY631756.1) contains a 1272 bp CDS encoding 423 amino acid residues, and a 966 bp 3′ untranslated region (UTR). The molecular weight of the corresponding porcine SLC35D3 protein is 44,653.9 Da and the isoelectric point is 6.94. Three potential O-glycosylation sites and 42 phosphorylation sites were found using functional site prediction algorithms. SLC35D3 does not appear to possess a signal peptide structure and is therefore unlikely to be secreted from the cell. In the predicted secondary structure, 37.12% of the amino acids participate in α-helixes, 7.8% in β-folds, and 33.57% in random coils (Fig. 1a). Eight transmembrane domains were predicted by TMHMM analysis (Fig. 1e). Analysis using ProtScale suggests that the protein has a distinct hydrophobic region (Fig. 1b). To determine the level of amino acid conservation in homologous proteins, a multiple sequence alignment analysis was performed (Fig. 1d), revealing highly conserved regions. SWISS-MODEL was used to predict the 3D structure (Fig. 1c). Homology modeling suggests that the structure of porcine SLC35D3 is not highly homologous to human 5i20.1.A (PDB: D7A5Q8). Together, these results provide a foundation for further study of the relationship between structure and function. Finally, we used neighbor-joining to construct a phylogenetic tree from the SLC35D3 protein sequence of the Yimeng Black pig and other mammals (Fig. 1f). SLC35D3 from the Yimeng Black pig has the highest homology to members of the genus *Ovis aries.*

### Expression of *SLC35D3* in porcine tissues from different breeds and ages

**Fig. 2:**
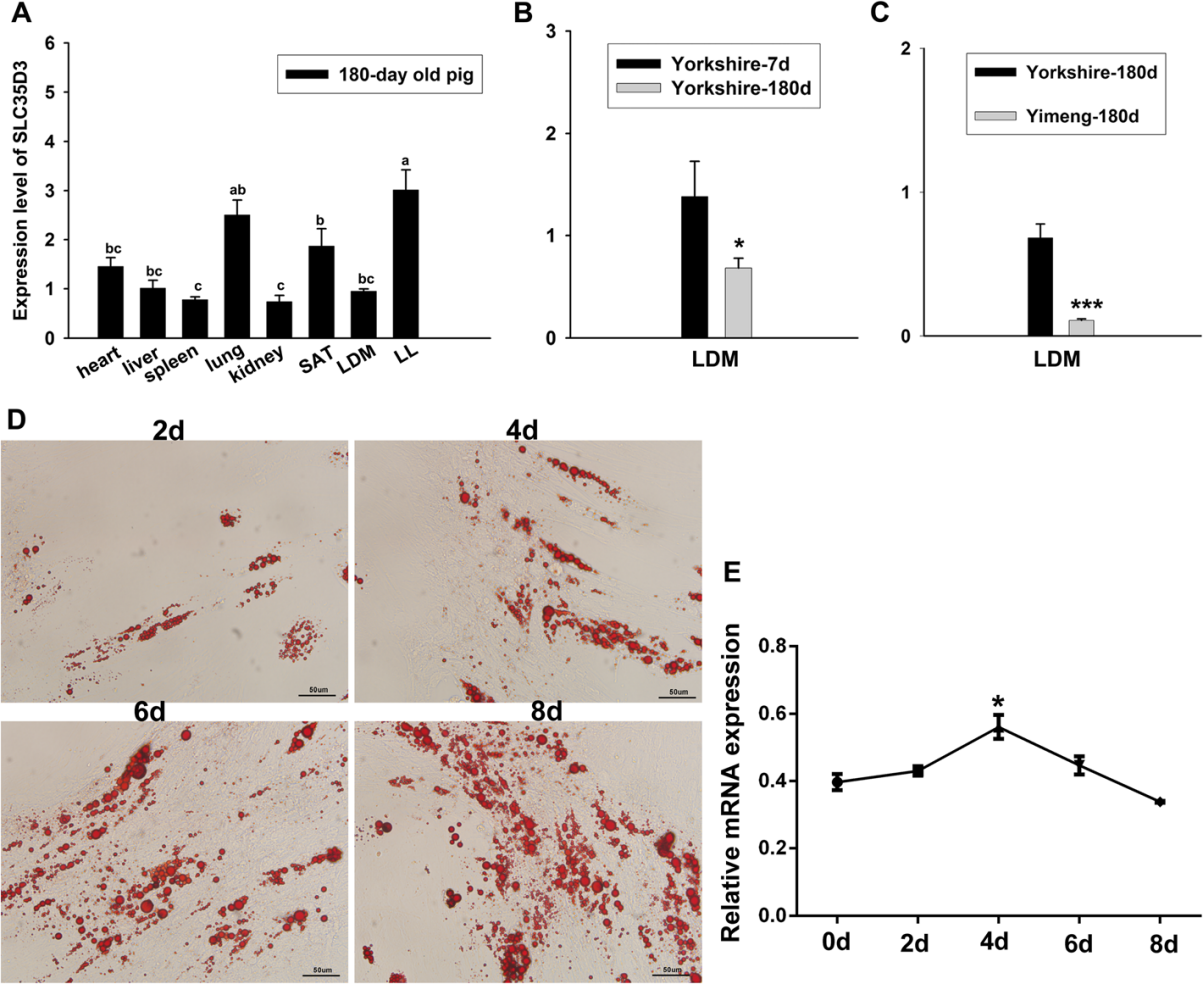
Expression profile in porcine tissue distribution at different stages, breeds and during intramuscular preadipocyte differentiation. **a** Expression of *SLC35D3* mRNA in porcine tissues from 180-day-old Yorkshire pigs. *18S* was used as an internal control. **b** *SLC35D3* mRNA expression levels in LDM from 7-day and 180-day old Yorkshire pigs. *18S* was used as an internal control. **c** *SLC35D3* mRNA expression levels in LDM from 180-day old Yorkshire and Yimeng Black pigs. *18S* was used as an internal control. **d** Oil Red O staining of porcine intramuscular adipocytes (scale bar, 50 μm). **e** *SLC35D3* mRNA expression during intramuscular preadipocyte differentiation. *GAPDH* as an internal control. Data are shown as means ± S.E.M. *n* = 3

*SLC35D3* mRNA levels in tissues from 180-day old Yorkshire pigs were quantified by RT-qPCR and normalized using *18S* RNAs (Fig. 2a). mRNA was isolated from liver, kidney, lung, heart, spleen, LDM, LF, and SAT. *SLC35D3* expression was high in lung, LF, LDM, and SAT, suggesting that *SLC35D3* may play an important role in adipose deposition.

We also compared *SLC35D3* expression in the LDM in Yorkshire and Yimeng Black pigs. *SLC35D3* expression was higher in Yorkshire 7-day old pigs than in 180-day old pigs (Fig. 2b); between the 180-day old Yorkshire and Yimeng Black pigs, expression was higher in the Yorkshire breed (Fig. 2c).

### Expression profile of *SLC35D3* during intramuscular preadipocyte differentiation

We performed RT-qPCR to monitor mRNA expression of *SLC35D3* and the marker genes *PPARγ*, *FASN,* and *FABP4,* in cultured preadipocytes that were undergoing differentiation. Cells were cultured for 2 days after reaching a density of 90%, when they had reached confluency, then were induced to differentiate. Cells were assayed 0, 2, 4, 6, and 8 days after the induction of differentiation. *SLC35D3* mRNA expression gradually increased to its maximum level at day 4, and then decreased thereafter (Fig. 2e). Lipid droplet accumulation increased throughout the 8-day experiment (Fig. 2d).

### Silencing of *SLC35D3* expression promotes adipogenesis during differentiation of porcine intramuscular preadipocytes

**Fig. 3:**
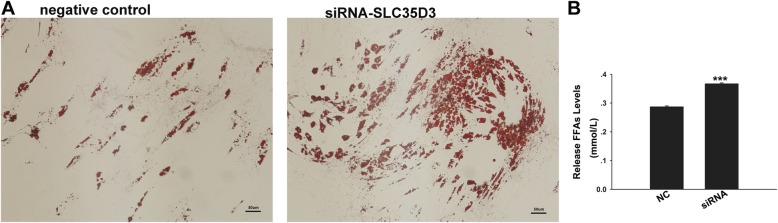
Knockdown of *SLC35D3* increased porcine intramuscular preadipocyte differentiation (**a**) The effects of *SLC35D3* silencing on lipid droplet accumulation in intramuscular adipocytes 8 days after the induction of differentiation (scale bar, 50 μm). **b** The effects of *SLC35D3* gene silencing on free fatty acid in culture medium of porcine intramuscular adipocytes. Data are shown as means ± S.E.M. *n* = 3. NC, negative control siRNA; siRNA, *SLC35D3*-siRNA

**Fig. 4:**
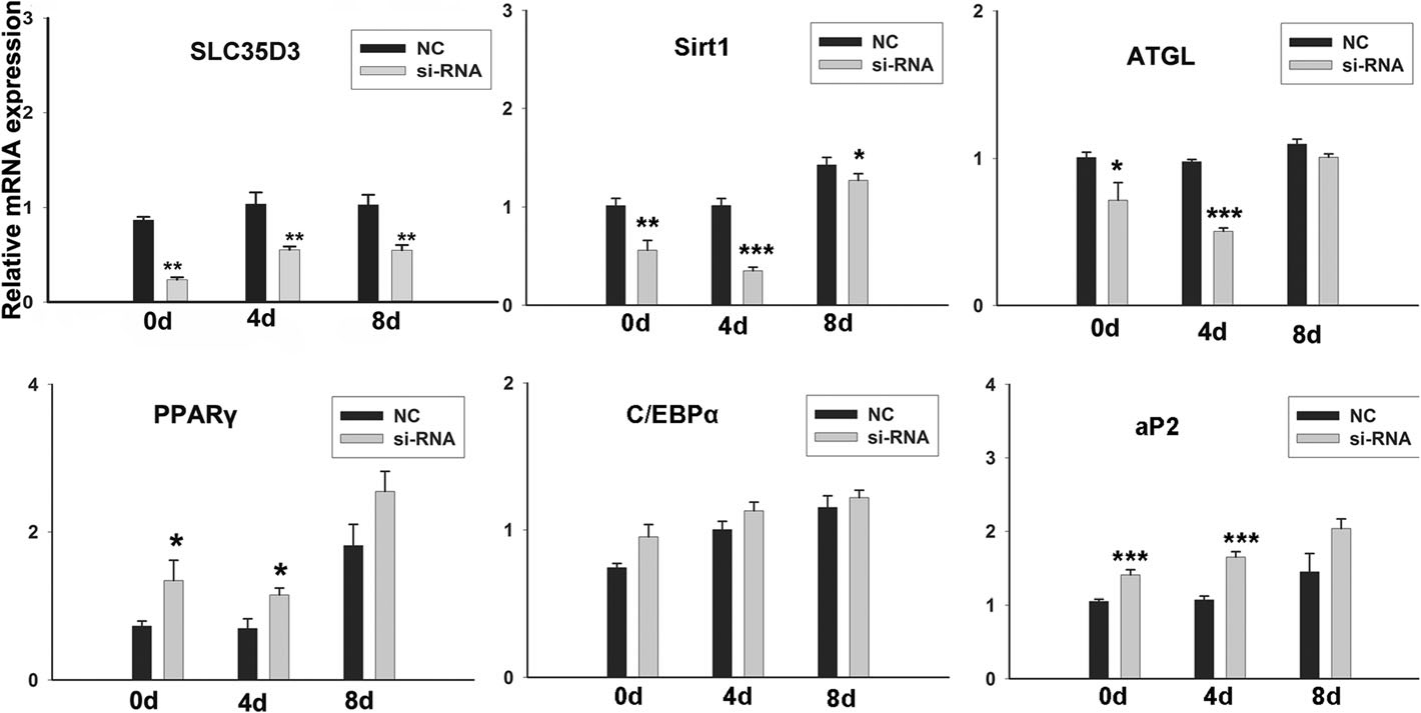
mRNA expression profile of *SLC35D3* and adipogenic marker genes during intramuscular adipogenesis by RT-qPCR. *GAPDH* is the internal control. Data are shown as means ± S.E.M. n = 3

The inhibition efficiency of four candidate siRNAs (siRNA 1–4) was 28, 41, 50, and 24% respectively; the most effective, siRNA3, was used to inhibit the expression of *SLC35D3* gene during intramuscular adipocytes differentiation. As demonstrated by Oil Red O staining of *SLC35D3* silenced porcine intramuscular preadipocytes, adipogenesis was greatly increased over control cells at 8 days post induction (Fig. 3a); the level of free fatty acid (FFA) released into the culture medium also increased (Fig. 3b). As expected, relative expression of *SLC35D3* decreased significantly in cells transfected with siRNA3 (Fig. 4). RT-qPCR was also used to detect the expression of five genes related to fat synthesis and catabolism (Fig. 4). Decreased expression was observed for *Sirt1* and *ATGL* at one or more time points. In mature adipocytes, *Sirt1* promotes fat mobilization through repression of *PPARγ* [20]. *ATGL* is expressed in many tissues, particularly adipocytes, where it promotes both basal and stimulated lipolysis [21]. In contrast, increased expression was observed for *PPARγ*, *C/EBPα,* and *aP2*, although the differences were not significant for *C/EBPα*. *PPARγ* is expressed in white and brown adipose tissue, though it’s expressed most highly in adipocytes and plays a key role in the regulation of adipogenesis, energy balance, and lipid biosynthesis [22]. *C/EBPα* is primarily expressed in fat, it is a key regulator at the adipogenic initiation stage, and it synergistically triggers adipocyte-specific gene expression with *PPARγ* after the growth arrest stage [23]. *aP2* is actively secreted from adipocytes, and is regulated by fasting- and lipolysis-related signals; circulating *aP2* levels are markedly elevated in obese mice and humans [24]. Overall, these results suggest that interference with *SLC35D3* gene expression promotes porcine intramuscular preadipocyte differentiation. We speculate that *SLC35D3* can inhibit the adipogenic process in porcine intramuscular adipocytes.

## Discussion

*SLC35D3,* a recently discovered gene, is involved in metabolic control, and is a candidate gene for MetS [16]. In this study we cloned, sequenced, and characterized the porcine *SLC35D3* gene. The porcine *SLC35D3* sequence is similar to that described in other animals. Based on predicted physicochemical properties, porcine SLC35D3 is a hydrophobic, multi-transmembrane protein. Further sequence analysis revealed the stability and conservation of *SLC35D3* gene structures.

We showed that *SLC35D3* can inhibit the adipogenic process in pig intramuscular adipocytes. Our results uncover a previously unknown role of *SLC35D3* in porcine adipogenesis, and suggest a fruitful area of research for improving the quality of porcine meat quality as well as its potential role in human cardiovascular diseases.

Essential in the study of mechanisms involved in fat deposition, is the study of IMF. IMF is a primary factor of meat quality, it is affected by fatty acid transport, fat anabolism, and catabolism. To date there are no studies examining expression of *SLC35D3* in the various tissues of pigs based on age or breed. Our results showed that the expression levels of *SLC35D3* were high in fat and LDM tissue. In LDM, expression is higher in 7-day old piglets than in 180-day old pigs. In addition, the level of gene expression is significantly different in Yorkshire and Yimeng Black pigs. The Yorkshire pig is a typical lean meat breed, it has less subcutaneous and intramuscular fat than the Yimeng Black pig. It is still unknow whether the expression level of *SLC35D3* is related to the fat deposition between the two pig breeds. During differentiation of porcine intramuscular preadipocytes, *SLC35D3* expression increased from day 0 to day 4 and then declined until day 8. This trend was also seen with the expression of the adipogenic marker genes. We therefore speculate that *SLC35D3* has important functions during adipocyte differentiation, however the underlying mechanism is still unknown. Adipogenesis is a complex programmed process, during which the early adipogenic transcription factors *C/EBPα* and *PPARγ* are activated, inducing the expression of adipogenic genes. These factors also play a critical role in the terminal differentiation of adipocytes in vitro, ultimately leading to the formation of mature adipocytes [25, 26]. *ATGL* initiates the process of triglyceride metabolism by hydrolyzing triglycerides into diacylglycerol and fatty acids [27, 28]. We found that reducing expression of *SLC35D3* in porcine intramuscular preadipocytes resulted in siginificantly higher expression of adipogenic marker genes such as *PPARγ* and aP2, while inhibiting the expression of lipid hydrolytic gene *ATGL*. However, the observed trends of *C/EBPα* were not significant. Consistent with these results, FFA levels increased in the culture medium of intramuscular preadipocytes during differentiation. These results together suggest that *SLC35D3* may be a specific transcription regulatory factor during porcine intramuscular preadipocyte differentiation.

## Conclusions

In summary, this is the first report describing the cDNA sequence of *SLC35D3* from the Yimeng Black pig. The gene contains a 1272 bp CDS that encodes 423 amino acids, and a 966 bp 3′ UTR. The deduced amino acid sequence of *SLC35D3* is highly similar (92–95%) to homologous proteins in other mammalian species. Phylogenetic analysis shows that pig *SLC35D3* has a close evolutionary relationship with the *Ovis aries* version of the gene. Gene expression analysis suggests that *SLC35D3* inhibits adipogenesis in pig intramuscular preadipocytes. Our results provide a basis for further studies on the function and regulation of *SLC35D3*.

## Methods

### Experimental animals and sampling

Three 180-day old Yorkshire pigs (average live weight 100 kg; range, 99–105 kg), three 7-day old Yorkshire pigs (average live weight, 1 kg; range, 0.8–1.3 kg), and three 180-day old Yimeng Black pigs (average live weight 100 kg; range, 90–110 kg) were purchased from the experiment farm at the Chinese Academy of Agricultural Sciences; all animals were female. Animals had been maintained under the same management conditions, fed the same food three times a day, and had access to water ad libitum. Animals selected for tissue collection were humanely euthanized by electrical stunning followed by exsanguination. Tissue samples from the liver, kidney, lung, heart, spleen, LDM, LF, and SAT were dissected from each pig, immediately frozen in liquid nitrogen, transported to the laboratory, and stored at − 80 °C until RNA extraction. Intramuscular preadipocytes were collected from the 7-day old piglets and cultured. Finally, stored at − 80 °C until use.

### RNA isolation and cDNA synthesis

Total RNA was extracted from tissues and cells using TRIzol reagent (Invitrogen, Carlsbad, CA, USA). RNA concentrations were determined by absorption spectrophotometry at 260 nm. First-strand cDNA synthesis was conducted with 2 μg of purified total RNA using a RevertAid First-Strand cDNA Synthesis Kit (Thermo Scientific, Waltham, MA, USA). RNA and cDNA were stored at − 80 °C and − 20 °C, respectively.

### PCR amplification of the internal fragment of *SLC35D3*

Forward (F) and reverse (R) primers (Table 1) were designed with the Primer Premier 5.0 application, using porcine *SLC35D3* (XM_013986971.1) as the reference gene sequence. The PCR reaction contained 1 μL of cDNA, 12.5 μL of 2× Es Taq Master Mix, 0.5 μL of each primer, and 10.5 μL of RNase-free water. PCR cycling conditions were 94 °C for 5 min, then 34 cycles of 94 °C for 30 s, 57 °C for 5 s, 72 °C for 26 s, followed by 72 °C for 10 min.

**Table 1:** Primer sequences and their use in this study

Primer name	Primer sequence (5′ → 3′)
F	TGCACCTACATCAACTCGG
R	TCATTTCTTCAGGGCTGTCT
UPM long	CTAATACGACTCACTATAGGGCAAGCAGTGGTATCAACGCAGAGT
UPM short	CTAATACGACTCACTATAGGGC
GSP3	GGTGAAGAGCATCGCCACCATCACGG
GSP5	CCCCTCGCCTCTTGCCCACCCTGCT
NGSP5	GCCTTCCCACCTCCTGACCCGCC
*SLC35D3*-F	CCTCAGCCTGCCTATGTACG
*SLC35D3*-R	CAGCGCTTGCTTTCTGGATG
*ATGL*-F	TTGCTGTCAACCAACCACTC
*ATGL*-R	TAATAGTGCTCTGAGGGCCG
*PPARγ*-F	AGGACTACCAAAGTGCCATCAAA
*PPARγ*-R	GAGGCTTTATCCCCACAGACAC
*Sirt1*-F	AACCGATGGAGAGTCCAGGT
*Sirt1*-R	TACCTCAGCGCCATGGAAAA
*aP2*-F	GAGCACCATAACCTTAGATGGA
*aP2*-R	AAATTCTGGTAGCCGTGACA
*C/EBPα*-F	CGATGCTCTTAGCTGAGTGT
*C/EBPα*-R	GGTCCAAGAATTTCACCTCT
*GAPDH*-F	AGGGCATCCTGGGCTACACT
*GAPDH*-R	TCCACCACCCTGTTGCTGTA
*18S*-F	CGTCTGCCCTATCAACTTT
*18S*-R	TTTCTCAGGCTCCCTCTC

### 5′ RACE and 3′ RACE

First-strand cDNA synthesis was accomplished using the SMARTer RACE 5′/3′ Kit (Takara, Dalian, China) according to the manufacturer’s protocol. 5′ RACE and 3′ RACE reactions were performed by nested PCR, using the *SLC35D3*-specific primers GSP5/3 and NGSP5 and the universal primers UPM long and UPM short (Table 1).

All PCR products, including the internal fragment and fragments generated by 5′ RACE and 3′ RACE, were subjected to agarose gel electrophoresis, then recovered using an agarose gel DNA Purification Kit (Tiangen, Beijing, China). The products were cloned into the pEASY-T1 vector (Trans, Beijing, China). Clones were submitted to Sangon Biotech Co., Ltd. (Shanghai, China) for nucleotide sequencing.

### Sequence analysis

Molecular weight and isoelectric point were predicted using Compute pI/Mw (http://us.expasy.org/tools/pi_tool.html). The secondary structure of the deduced amino acid sequence was predicted by SOPMA (http://npsa-pbil.ibcp.fr/) [29]. Phylogenetic analyses were performed using MEGA 5.1, applying the neighbor-joining method [30]. Amino acid sequences from different species were aligned using DNAMAN V6 (LynnonBiosoft, Los Angeles, CA, USA) [31]. SWISS-MODEL was used to model 3D protein structure [32, 33]. TMHMM Server v2.0 program (http://www.cbs.dtu.dk/services/TMHMM/) was used for protein transmembrane Structure analysis. ExPASy ProtScal (http://web.expasyorg/protscale/) was used for hydrophobic analysis. The signal peptide was predicted using SignalP (http://www.cbs.dtu.dk/services/SignalP/) [34]. N-glycosylation and O-glycosylation sites were predicted using NetNGlycears 1.0 (http://www.cbs.dtu.dk/services/NetNGlyc/) and NetOGlycubles 3.1 (http://www.cbs.dtu.dk/services/NetOGlyc/), respectively [35].

### RT-qPCR for expression profile analysis

Primers for *SLC35D3*, *Sirt1* (NM_001145750.2), *ATGL* (EF583921.1), *PPARγ* (NM_214379), *C/EPBα* (XM_003127015), and *aP2* (AJ555153.1) were designed using Primer Premier 5.0 (Premier Biosoft International, Palo Alto, CA). Relative mRNA levels were normalized against *GAPDH* and *18S* expression. The PCR reaction contained 7.2 μL of 2× SYBR Premix Ex Taq (Takara, Dalian, China), 0.3 μL of each primer, 1 μL of cDNA, 0.3 μL of Dye II, and sterile water to a final volume 15 μL. PCR cycling conditions were: 95 °C for 5 min, followed by 40 cycles at 95 °C for 5 s and 60 °C for 34 s. Finally, a dissociation step was performed at 95 °C for 15 s, 60 °C for 1 min, and 95 °C for 15 s. All samples were amplified in triplicate, and the mean was used for further analysis. Amplification of target genes was determined using the 2^-ΔΔCt^ method.

### Isolation and culture of intramuscular preadipocytes

LDM was collected from 7-day old piglets under aseptic conditions. Tissue samples were washed 3 times in PBS containing 1% penicillin and streptomycin, and cut into small pieces (approximately 1 mm^3^). Tissue pieces were digested in 0.1% type I collagenase (Invitrogen, Carlsbad, CA, USA) for 1 h at 37 °C, then filtered through 400 mesh filters. The filtrates were centrifuged for 5 min at 1500 rpm/min. Cell pellets were washed with PBS then centrifuged again for 5 min at 1500 rpm/min, repeat 3 times. The preadipocytes were resuspended in DMEM/F12 containing 10% fetal bovine serum (FBS) (Sigma, St. Louis, MO, USA) and 100 U/mL penicillin and streptomycin, then seeded into 6-well plates at a density of 5 × 10^4^ cells/cm^2^, and incubated at 37 °C in a humidified 5% CO_2_ atmosphere. Culture medium was changed every two days.

### siRNA design and transfection

Gene-specific siRNAs (Table 2) for *Sus scrofa SLC35D3* was synthesized based on our cDNA sequence; these were designed and synthesized by Gene Pharma Co., Ltd. Preadipocytes at 70–80% confluence were transfected with a negative control siRNA (20 nM) or *SLC35D3*-siRNA (20 nM) using Lipofectamine 2000 in OPTI-MEM, according to the manufacturer’s protocol. 48 h after transfection, culture medium was replaced with DMEM/F12 supplemented with 10% FBS and IBMX-DEX-insulin (0.5 mmol/L IBMX, 1 mol/L DEX, 5 mg/mL insulin) to induce differentiation. Cells were incubated in this medium for 48 h, then the culture medium was changed to DMEM/F12 with 10% FBS and 5 mg/mL insulin, this medium was changed every 2 days. Total RNA was extracted from cells on days 0, 4, and 8.

**Table 2:** siRNA sequences

name	seqence(5′ → 3′)
negative control	Sense 5′-UUCUCCGAACGUGUCACGUTT-3′ Antisense 5′-ACGUGACACGUUCGGAGAATT-3’
*SLC35D3*-siRNA1	Sense 5’-UCAUCAGCCGCUACCAGUUTT-3′ Antisense 5′-AACUGGUAGCGGCUGAUGATT-3’
*SLC35D3*-siRNA2	Sense 5’- GCAUCUUCGUGGCCUGUAUTT −3′ Antisense 5′- AUACAGGCCACGAAGAUGCTT − 3’
*SLC35D3*-siRNA3	Sense 5’-GCCCACCUCUCUAUUCAUUTT-3′ Antisense 5′-AAUGAAUAGAGAGGUGGGCTT-3’
*SLC35D3*-siRNA4	Sense 5’-GGAAGUGUGGCGGUUAGUUTT-3′ Antisense 5′-AACUAACCGCCACACUUCCTT-3’

### Oil red O staining

Oil Red O staining was conducted as described previously [36].

### Measurement of cellular lipid metabolites

Preadipocytes were induced, as described above, for 8 days, then culture medium was collected and submitted to North Life Science Co., Ltd. (Beijing, China) for free fatty acid analysis. The FFA measurements were conducted according to the manufacturer’s protocol provided in the free fatty acid analysis kit (Njjcbio, Nanjing, China).

### Statistical analysis

Data was analyzed using the SPSS v22.0 (SPSS, Chicago, IL), and one-way analysis of variance was used to assess the significance of experimental results. All data are presented as the means ± standard error of the mean. Differences were considered significant at a *P* value of < 0.05 (*), < 0.01 (**), or < 0.001 (***).

## Data Availability

All gene sequences in this study were deposited in NCBI. *SLC35D3* (KY631756.1), *Sirt1* (NM_001145750.2), *ATGL* (EF583921.1), *PPARγ* (NM_214379), *C/EPBα* (XM_003127015), and *aP2* (AJ555153.1).

## Abbreviations

CDS: Coding sequence
FBS: Fetal bovine serum.
FFA: Free fatty acid
IMF: Intramuscular fat
LDM: Longissimus dorsi muscle
LF: Leaf fat
MetS: Metabolic syndrome
SAT: Subcutaneous adipose tissue
SLC35: Solute carrier family 35
UTR: Untranslated region
